# Leiomyosarcoma in the extremities and trunk wall: systematic review and meta-analysis of the oncological outcomes

**DOI:** 10.1186/s12957-022-02584-4

**Published:** 2022-04-18

**Authors:** Sudhir Kannan, Han Hong Chong, Bryan Chew, Jay Dee Ferguson, Euan Galloway, Thomas McCulloch, Kenneth S. Rankin, Robert U. Ashford

**Affiliations:** 1 Health Education England, Waterfront 4 Goldcrest Way, Newburn Riverside, Newcastle upon Tyne, NE15 8NY UK; 2 Newcastle upon Tyne Hospitals NHS Foundation Trust, Freeman Rd, High Heaton, Newcastle upon Tyne, NE7 7DN UK; 3 Newcastle University, Newcastle upon Tyne, NE1 7RU UK; 4 University Hospitals of Leicester NHS Trust, Gwendolen Rd, Leicester, LE5 4PW UK; 5 Nottinghamshire University Hospitals, City Hospital, Hucknall Rd, Nottingham, NG5 1PB UK

**Keywords:** Leiomyosarcoma, Soft tissue, Trunk wall, Extremities, Outcomes

## Abstract

**Background:**

Leiomyosarcomas are aggressive malignancies which can occur on the trunk and extremities whose pathogenesis is poorly understood. We aim to quantify the prognostic impact of various clinical and pathological markers on survival and recurrence of leiomyosarcomas.

**Methods:**

We conducted a systematic review as per PRISMA protocol. Survival, local recurrence, and metastasis were the outcome measures. Data were extracted from the studies for the outcome variables; the resultant odds ratios (OR) and 95% confidence interval (CI) were used for the synthesis of a forest plot.

**Results:**

Our search revealed thirteen studies comprising 1380 patients. Seven of these 13 publications were since 2012. Our analysis showed that tumor size larger than 5 cm adversely affected the outcome with an *OR* 3.39 (2.26–5.10, *p* < 0.01). Other factors which reduced the overall survival were positive margins of excision *OR* 2.12 (1.36–3.32, *p* < 0.01). A reduced risk of metastasis has strongly associated the use of radiotherapy with *OR* 10.84 (4.41–26.61, *p* < 0.01). Only a few studies analyzed the impact of factors on local recurrence.

**Conclusions:**

Size larger than 5 cm and positive margins of excision are associated with poor overall survival. In comparison, the use of adjuvant radiotherapy was associated with a lower metastatic rate. There is a need for methodically high-quality studies with more uniform study design and reporting to evaluate the impact of various risk factors on local recurrence and metastases.

**Level of evidence:**

Level 1 Prognostic

**Supplementary Information:**

The online version contains supplementary material available at 10.1186/s12957-022-02584-4.

## Background

Soft tissue sarcomas (STS) are a heterogeneous group of neoplasms of mesenchymal origin. There are major differences in behavior which need to be accounted for when assessing treatment effects. Leiomyosarcoma (LMS) is a subtype of STS with pure smooth muscle differentiation, comprising approximately 25% of all soft tissue sarcomas. LMS can arise from any area of smooth muscle in the body and any site where there is a blood vessel. The retroperitoneum or intra-abdominal region accounts for 35% of tumors, uterus accounts for 30% of LMS, extremities about 19%, and trunk approximately 16% [1].

Immunohistochemistry, in addition to histopathology, is commonly conducted in sarcoma centers as part of the LMS investigation. These markers are not prognostic, and no therapeutic agents targeting them have been developed to date [2]. The size and precise primary anatomical site of the tumor, as well as its depth in the soft tissues and proximity to vital structures such as major nerves and blood vessels, are all important factors to consider. This will have an impact on the surgical plan, which will have an impact on margins and potentially oncological outcome.

It has been suggested in some studies that uterine and extrauterine LMS may reflect distinct disease biology [3]. There are, however, both clinical and molecular studies which have failed to demonstrate any absolute difference between the two disease groups [4]. Apart from confusion surrounding molecular pathogenesis, accurate prediction of clinical behavior of these tumors has proven to be difficult as there are no universally accepted prognostic factors [5].

Historically, studies have included multiple STS combined in their analysis. However, the subtypes of STS are heterogenous entities. There are only a few articles which have performed variable analysis investigating the prognostic impact of various clinicopathological factors for LMS involving trunk wall and extremities [6–8]. Hence, we aimed to systematically review the literature and quantify the prognostic impact of various clinicopathological factors for overall survival, local recurrence, and metastases of LMS involving only extremities and trunk wall. To our knowledge, this is the first systematic review of LMS involving limb and trunk wall exclusively.

## Materials and methods

The literature search in the systematic review was performed according to the Preferred Reporting Items for Systematic Reviews and Meta-Analyses (PRISMA) protocol (Supplementary 1). This study is a Newcastle University research project with the research ID (Newcastle University project approval: 8391 S, record ID 29321). Prior to the review, the Cochrane and PROSPERO databases were searched to ensure that no previous similar reviews had been performed. The registration ID of this current meta-analysis on PROSPERO is CRD42022316227, and the link is https://www.crd.york.ac.uk/prospero/display_record.php? RecordID=316227.

The electronic databases MEDLINE and EMBASE were searched for eligible studies from their inception up to September 2021. The primary terms for literature search were “Leiomyosarcoma AND trunk wall,” “Leiomyosarcoma AND extremities,” “Leiomyosarcoma of trunk wall OR extremities,” “Soft tissue sarcoma of the trunk wall OR extremities,” and “Leiomyosarcoma AND soft tissues” (full search strategy as Supplementary 2).

### Selection of studies

After eliminating duplicate research, four authors (SK, HHC, BC, and JDF) assessed the titles and abstracts of the remaining papers using the following criteria: (1) English-language literature or an acceptable English translation for non-English language studies; (2) study design: comparative or observational (randomized, prospective, or retrospective) studies; (3) population: human over the age of 16 with LMS of the trunk wall and extremities; and (4) outcome: survival, local recurrence, and metastasis. In the event of a disagreement, the main author (SK) and senior authors (KSR, RUA) arbitrated. In addition, potential research was examined in the reference lists of included studies. The full-text publications were screened using the same method as the abstracts.

### Data extraction

Three reviewers (SK, BC, and JDF) independently extracted data from the selected full-text articles, with oversight from the senior authors (KSR, RUA) to address any discrepancies. This included the author, year of publication, sample size, demographic characteristics, pathological characteristics, management, outcome, and complications. There was sufficient information reported in the manuscripts to extract the required data for the study.

### Outcome measures

The interested oncological outcomes to establish were survival, local recurrence, and metastases. The odds ratio (OR) or hazard ratio (HR) was calculated manually using the published data in the articles by 2 × 2 contingency tables. The resultant OR and 95% confidence interval (CI) were used for the synthesis of a forest plot.

### Quality of studies

The overall quality of the studies was evaluated by four authors (SK, HHC, BC, and JDF) using a modified Newcastle-Ottawa scale for cross-sectional studies [9]. Furthermore, we have used quality assessment guidelines for prognostic studies published by Hayden et al. to assess the methodical quality of the studies included in our meta-analysis [10]. Efforts were made to remove all potential duplicated data across included studies and include all studies published to date. Funnel plots were used to visually inspect the relationship between sample size and treatment effects for the two groups and assess publication bias.

### Data synthesis and analysis

Nordic Cochrane Centre, Copenhagen, Denmark) to pool the data, and *p* < 0.05 was considered significant. Data were pooled using inverse variance method after calculating the relevant OR and CI. A random-effect model was used to allow equal representation from each study. Forest plots were formulated to illustrate the relative strengths and significance of the studies.

## Results

**Fig. 1:**
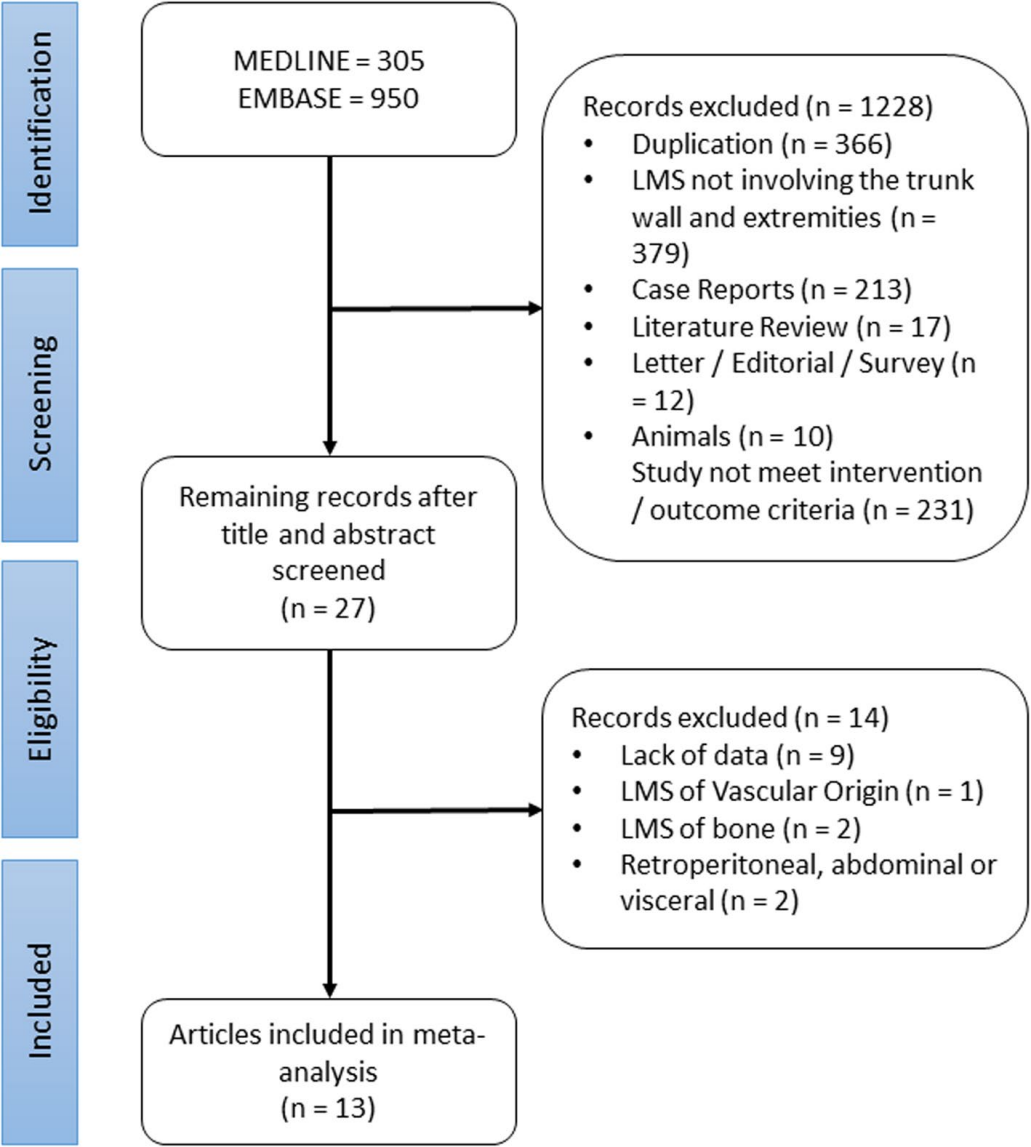
PRISMA flowchart studies selection. LMS, leiomyosarcoma

One-thousand two-hundred and fifty-five studies were identified for potential eligibility, 305 from MEDLINE and 950 from Embase. Following the screening process, 13 articles were included in the final meta-analysis (Table 1). The bibliography of these articles was manually checked to identify any missing studies (Fig. 1).

**Table 1:** Characteristics of studies included in the meta-analysis

Study/year	***N*** =	Age	Gender	Grade	Size in cm	Location in relation to fascia	Excision margin	Adjuvant treatment	LR	DM	Survival data
Hashimoto et al. 1986^a^ [11]	24	Median 55	M: 8 F: 16	1: 6 2: 14 3: 5	≤ 5: 8 > 5: 12 NR: 3	Above: 11 Below: 13	Marginal: 21 Wide: 2 Amputation: 1	CT: 5 RT: 2	11	9	5-year survival 61%
Gustafson et al. 1992 [1]	48	Median 65	M: 26 F: 22	1: 2 2: 4 3: 17 4: 25	≤ 5: 24 > 5: 24	Above: 21 Below: 27	Intralesional: 1 Marginal: 21 Wide: 26	CT: 8 RT: 11	10	20	5-year survival 64%
Miyajima et al. 2002^b ^[8]	267	Median 58	M: 125 F: 142	1: 110 2: 115 3: 42	≤ 5: 43 > 5: 143 NR: 83	Above: 105 Below: 162	Resection: 264 Unresectable: 3	0	NR	NR	AJCC 5-year survival Stage I 78% Stage II 49% Stage III 35% Stage IV 0%
Farshid et al. 2002 [4]	42	Mean 60	M: 21 F: 21	1 and 2: 20 3: 18 NR: 4	≤ 4: 18 > 4: 20 NR: 4	Above: 15 Below: 27	Intralesional: 4 Marginal: 13 Wide: 21 Indeterminate: 4	CT: 2 RT: 11	3	17	Median survival 35 months
Massi et al. 2004 [7]	42	Median 67.5	M: 20 F: 22	1: 1 2: 12 3: 29	≤ 5: 9 > 5: 33	Above: 1 Below: 41	Intralesional: 5 Marginal: 4 Wide: 33	CT: 9 RT: 21	9	16	5-year survival 32.6%
Svarvar et al. 2006^c^ [12]	225	Median 70	M: 104 F: 121	1: 6 2: 33 3: 75 4: 108 NR: 3	≤ 5: 129 > 5: 91	Above: 137 Below: 88	Intralesional: 21 Marginal: 69 Wide: 129 No surgery: 6	CT: 11 RT: 64	NR	76	5-year survival 64%
Tsiatis et al. 2009 [13]	28	Mean 59	M: 14 F: 14	1: 3 2: 6 3: 19	NR	Below: 28	NR	RT: 20	NR	NR	Median survival 35 months
Abraham et al. 2012^d^ [14]	115	Mean 58	M: 48 F: 67	1: 10 2: 25 3: 80	≤ 5: 31 > 5: 84	Above: 20 Below: 95	Intralesional: 17 Marginal: 15 Wide: 57 No surgery: 26	CT: 45 RT: 45	31	70	5-year survival 57%
Gladdy et al. 2013^e^ [6]	353	Median 57	M: 196 F: 157	High: 265 Low: 88	≤ 5: 155 > 5: 194	Above: 96 Below: 257	Intralesional/marginal: 64 Wide: 289	NR	46	109	5-year survival 75%: extremity 81%: trunk
Farid et al. 2013^f^ [15]	46	Median 55	M: 23 F: 23	NR	NR	NR	Intralesional/marginal: 12 Wide: 10	RT: 19	2	12	Mean survival 40.5 months
Gordon et al. 2014 [16]	47	Mean 55.3	M: 24 F: 23	1: 2 2: 5 3: 35 NR: 5	< 10: 16 > 10: 31	Above: 17 Below: 24	Radical: 43 Palliative: 1 No surgery: 3	NR	6	32	NR
Worhunsky et al. 2015 [3]	48	Median Limbs: 60 Trunk: 55	Limbs M: 18 F: 8 Trunk M: 7 F: 15	1/2: 29 3: 19	≤ 5: 29 > 5: 19	Above: 14 Below: 34	Intralesional/marginal: 8 Wide: 40	NR	6	12	NR
Shoushtari et al. 2016^g ^[17]	215	Mean 56.4	M: 80 F: 135	1: 7 2: 5 3: 176 NR: 27	< 5: 34 ≥ 5: 161	Above: 5 Deep: 196 NR: 14	Intralesional/marginal: 48 Wide: 95 No surgery: 47 NR: 25	NR	NR	113	NR

*AJCC *American Joint Committee on Cancer staging, *cm *centimeter, *CT *chemotherapy, *DR *distant metastasis, *F *female, *grading *French Federation of Cancer Centers (FNCLCC) grading system, *LR *local recurrence, *M *male, *N *total sample size, *NR *not reported, *RT *radiotherapy

^a^one case reported anatomical location on the head, which was excluded from the sample size and assumed to be marginal excision. There is insufficient information on adjuvant chemo/radiotherapy, and no recurrence has been reported

^b^location of leiomyosarcoma was not specified — assumption made to be trunk and extremities

^c^six included cases reported anatomical location on the head — information could not be retrieved individually from the pooled summary features

^d^fifty-four included cases reported anatomical location in the retroperitoneum or others — information could not be retrieved individually from the pooled summary features

^e^one-hundred and forty-four included cases reported anatomical location in the abdominal or retroperitoneum—information could not be retrieved individually from the pooled summary features

^f^thirteen included cases reported anatomical location in the gastrointestinal tract, genitourinary, and others—information could not be retrieved individually from the pooled summary features

^g^one-hundred and sixty-one included cases reported anatomical location in the abdomen and others—information could not be retrieved individually from the pooled summary features

All included studies were retrospective analysis. The total number of patients included was 1500, of which 1121 had a diagnosis of LMS of trunk wall or extremities. Desired data were available only in 1380 patients. The median age at presentation was 58 years (range 55–70 years). A total of 52% of the patients (786/1500) were females, and 48% (714/1500) were males. A total of 83% (1020/1230) of tumors occurred in extremities; 17% (210/1230) occurred in the trunk wall. With regard to size, 65% of the tumors were more than 5 cm (899/1379) and 44% (654/1484) were less than 5 cm. A total of 68% (992/1454) of tumors were deep to the deep fascia.

Most of the articles included in this study used the French Federation of Cancer Centers Sarcoma Group (FNCLCC) grading (11/13). However, all the articles have expressed their results as a 2-, 3- or 4-tier grading—80.2% (1106/1380) of the tumors were high grade (2).

Treatment data were available for 953 patients. These data showed that 30.3% (289/953) of tumors were excised with positive margins; this included 40 intralesional excisions. A total of 250/289 positive margins were unexpected following excision with documented curative intent surgery; there was no documentation regarding the intent of surgical excision in the rest of the patients. Data on intralesional excisions were available only in three articles [1, 12, 14], and one article [6] is distinguished between microscopically or macroscopically positive margins.

**Fig. 2:**
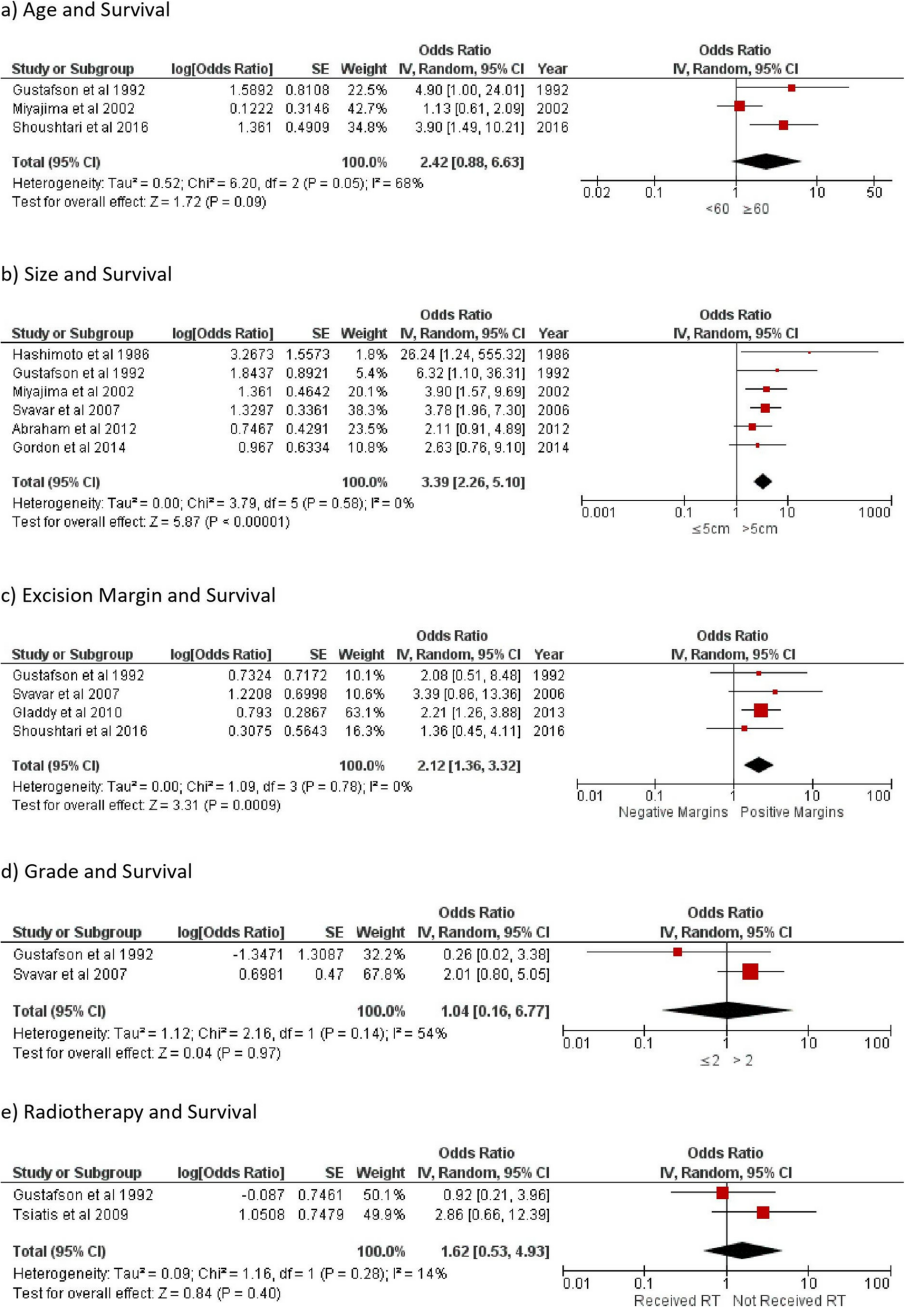
Forest plot showing the odd ratio of survival rate in leiomyosarcoma in relation to risk factors of **a** age, **b** size of tumor, **c** excision margin, **d** tumor grade, and **e** adjuvant radiotherapy. SE, standard error; CI, confidence interval; *I*^2^, level of heterogeneity; RT, radiotherapy

The overall metastasis rate was 40% (486/1205). Analysis showed that the median earliest time to local recurrence was 6 months (1–22.8 months), and the median earliest time for distant recurrence (metastases) was 12 months (5–22.5 months). The lung was the most common site for metastases. Pooled meta-analysis for disease-specific survival rate and metastasis is presented in Fig. 2.

### Disease-specific survival (Fig. 2)

All thirteen studies included in this review have published the disease-related mortality data. Svavar et al. [12] also published death due to other causes and unidentifiable causes in addition to disease-specific mortality. Three articles [1, 8, 17] (369 patients) published data on the impact of age of occurrence of the tumor on survival. Our analysis suggested that age had no association to higher mortality (*OR* 2.42; 95% *CI*: 0.88–6.63; *p* = 0.09). There were six articles [1, 8, 11, 12, 14, 16] (665 patients) with published data on the effect of tumor size on survival. Tumors larger than 5 cm had a higher risk of mortality with an OR of 3.39 (95% *CI*: 2.26–5.01; *p* < 0.01).

A further two articles [1, 12] (267 patients) published data on the effect of the grade of the tumor on survival. Our analysis showed that the tumor grade (of > 2) did not significantly impact the risk of death (*OR* 1.04; 95% *CI*: 0.16–6.77; *p* = 0.97).

Only one [17] article published the effect of the depth of tumor invasion in relation to fascia layer on survival for LMS affecting the trunk wall and extremities. Gladdy et al. [6] have published results for the impact of grade on survival; it applies to LMS in general rather than being site-specific. Hence, we have not combined data from these two articles for analysis.

Four articles [1, 6, 12, 17] comprising 530 patients published data on the impact of the margin of excision on survival. The data from these articles showed that the OR of positive surgical margins leading to poor survival is 2.12 (95% *CI*: 1.36–3.32; *p* < 0.01).

Two articles [1, 13] (76 patients) have published data on the effect of adjuvant radiotherapy (RT) on survival (31 patients received RT and 45 patients received no RT). The limited data from these studies suggest that radiotherapy had no impact on survival (*OR* 1.62; 95% *CI*: 0.57–4.55; *p* = 0.36). The role of chemotherapy and its impact on survival could not be assessed due to lack of data. Similarly, only one study [13] analyzed the impact of induction radiotherapy on survival.

### Development of distant metastases (Fig. 3)

Six out of 13 articles [4–7, 18, 19] included in the review have published data on metastases detected during follow-up. In addition, Abraham et al. [14] recorded data on metastasis at presentation, but they have excluded these from the final analysis. Four out of six studies [4, 6, 13, 14] published data on the effect of size larger than 5 cm on metastases for LMS affecting extremities and trunk wall, with the pooled data involving 350 patients. Further analysis revealed that the tumor size had no significant impact on the risk of metastasis (*OR* 1.41; 95% *CI*: 0.28–7.04; *p* = 0.68).

**Fig. 3:**
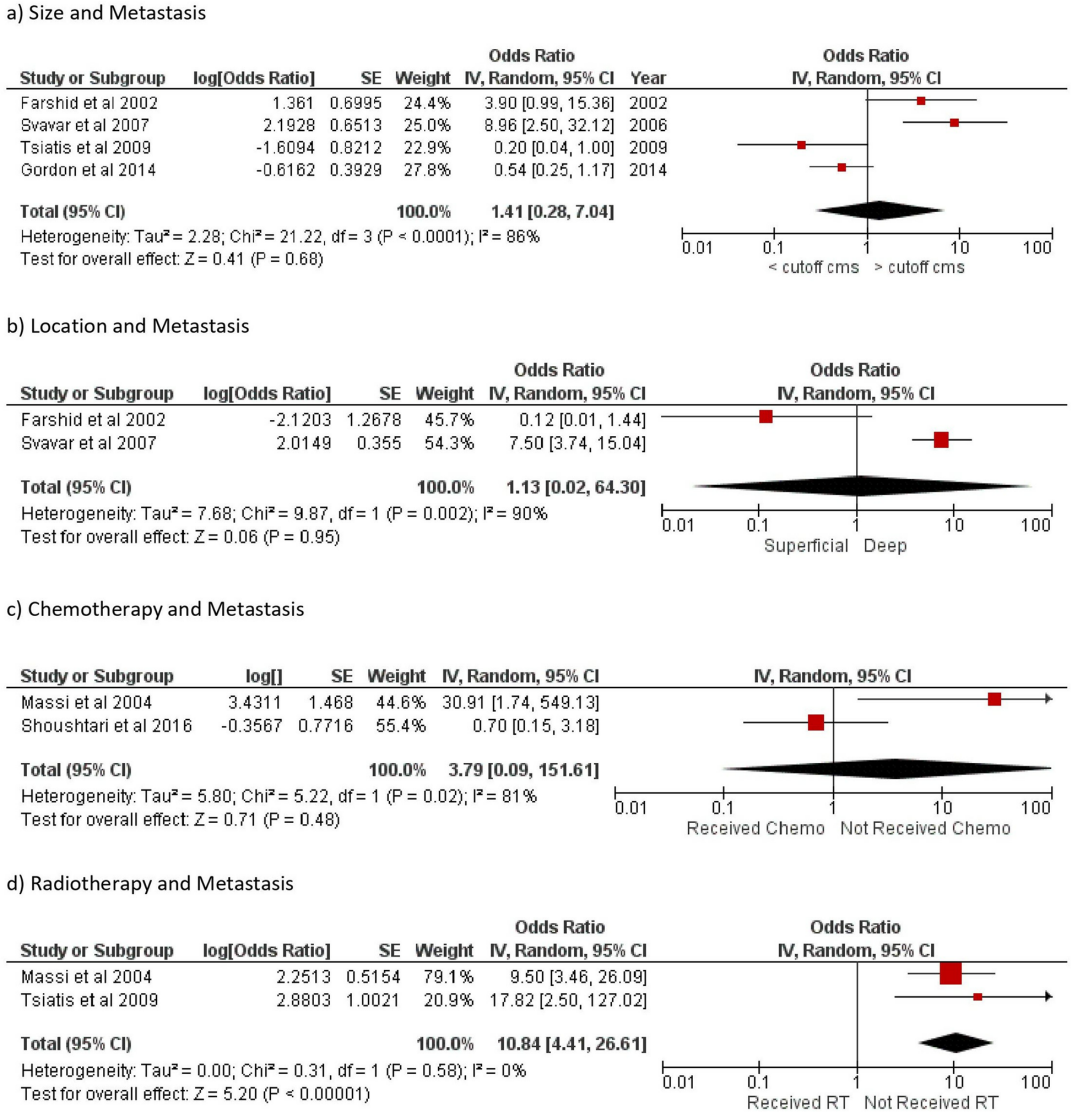
Forest plot showing the odd ratio of distant metastasis in leiomyosarcoma in relation to risk factors of **a** size of tumor, **b** location, **c** adjuvant chemotherapy, and **d** adjuvant radiotherapy. SE, standard error; CI, confidence interval; *I*^2^, level of heterogeneity; RT, radiotherapy

Similarly, two articles [4, 11] (189 patients) published data of the effect of the location of the tumor on metastases. On analyzing the data from these studies, we found that the tumors location has no impact on incidence of metastasis (*OR* 1.13; 95% *CI*: 0.02–64.30; *p* = 0.95).

Three articles [7, 13, 17] have published the impact of adjuvant treatment on occurrence of metastasis. Adjuvant radiotherapy [7, 13] (41 patients) showed a significant association with reduced odds of metastasis (*OR* 10.84; 95% *CI*:4.41–26.61; *p* = 0.00001). Two articles [7, 17] (98 patients) explored the effect of chemotherapy on metastasis. The data analysis suggested that chemotherapy did not make a difference to the overall survival (*OR* 3.79; 95% *CI*: 0.09–151.61; *p* = 0.48).

### Local recurrence

Only 2 out of 13 studies [6, 7] included in our analysis published the rate of local recurrence, but there were no results for the impact of the clinical or pathological factors on local recurrence.

Heterogeneity and risk of bias analysis

There is a wide diversity across the studies in clinical and methodological reporting. In the overall analysis, variable heterogeneity was reported, with an *I*^2^ value of 0–90%. For survival analysis, the heterogeneity is low to moderate, with an *I*^2^ value of 0–68%. On the other hand, the heterogeneity for metastasis analysis is moderate to substantial, with an *I*^2^ value of 0–90%. The inclusion of non-randomized controlled studies leads to different weighted data in analysis; hence, a random effect model was used to balance out the weight of each article and allow equal representation. None of the studies was related; so, no patients duplication was expected in the pooled analysis. The quality assessment of each study is summarized in Supplementaries 3 and 4. Overall, the articles included were deemed as moderate to high quality for non-randomized controlled studies.

**Fig. 4:**
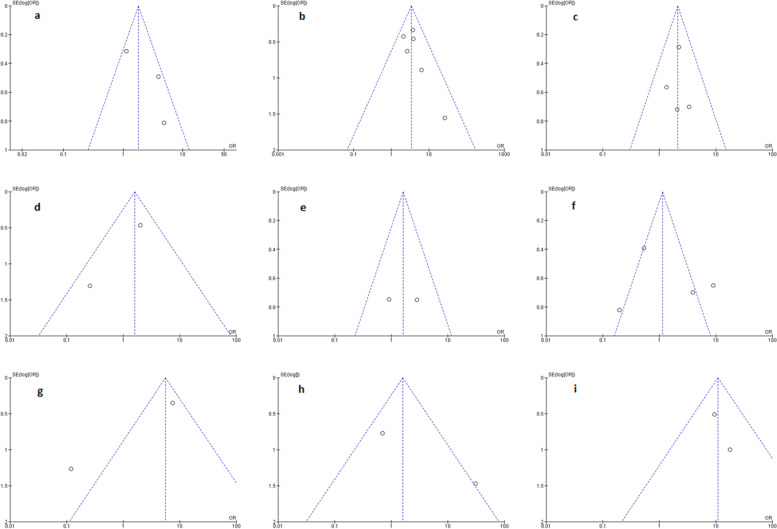
Funnel plot for publication bias assessment. **a** Age vs survival rate, **b** size vs survival rate, **c** excision margin vs survival rate, **d** tumor grade vs survival rate, **e** adjuvant radiotherapy vs survival rate, **f** size of tumor vs distant metastasis, **g** location vs distant metastasis, **h** adjuvant chemotherapy vs distant metastasis, and **i** adjuvant radiotherapy vs distant metastasis

We created a funnel plot for each comparison (Fig. 4) to assess the potential publication bias. Eight out of nine plots were inverted and funnel-shaped with bilateral symmetry, indicating a low-risk publication bias. The effect of size on survival rate was subjected to asymmetry of funnel plot (Fig. 4b), but we do not believe this contributes to publication bias or heterogeneity because most of this area contains regions of high importance.

## Discussion

Our findings suggest that a size greater than 5 cm and a positive excision margin may be independent prognostic indicators for mortality. According to the existing literature, excision margins, size, and deep origin may all be independent prognostic variables that increase the likelihood of local recurrence. Limited evidence on the impact of adjuvant treatment revealed that radiotherapy could be an independent factor in reducing the risk of metastasis. We found that only 2 [6, 7] articles specifically studied the impact of factors affecting local recurrence. Massi et al. [7] found out that only the type of excision was an independent predictor of decreased relapse. In their series, they reported that 7 out of 9 patients who developed local recurrence had marginal or intralesional excision. Furthermore, in all these patients, the first local recurrence was treated with surgical excision. Similarly, Gladdy et al. [6] found that size and margin status were independent predictors of both local and distant recurrence for LMS in general (no site-specific data).

There were two articles which focused on survival as their primary outcome variable. These were by Gustafson et al. and Gladdy et al., with the latter including tumors involving trunk wall and extremities [1, 6]. Gustafson et al [1]. concluded that age over 60 years old and intravascular invasion were independent risk factors for death resulting from the tumor. Their multivariate analysis showed that other factors like DNA aneuploidy and tumor necrosis were associated with poor prognosis but did not reach any statistical significance. On the other hand, Gladdy et al. [6] found that high grade and size greater than 10 cm were significant independent predictors of disease-specific survival for LMS of extremity, abdominal, retroperitoneal, and trunk wall tumors. Some findings of these studies are borne out by the overall findings of our systematic review, i.e., tumor size more than 5 cm had poor survival. Apart from Gladdy et al. and Gustafsson et al., Miyajima et al. also have studied the impact of various prognostic factors on survival. In the study conducted by Miyajima, only tumor size and AJCC staging were the prognostic factors independently predicting poor survival. However, Miyajima et al. did not specify the anatomical location of the tumor, although it is inferred in the discussion that retroperitoneal sarcomas were excluded [8].

Only three articles studied the prognostic significance of various factors with the development of distant metastases as their outcome. These studies were by Farshid et al [4]., Svavar et al. [12], and Gladdy et al. [6] Of these three studies, only the latter two have explicitly mentioned the factors which affect the development of metastases [4, 11]. On the other hand, Gladdy et al. have expressed the impact of factors on recurrence, which includes both local recurrence and metastases [6]. It is important to note that Farshid et al. excluded cutaneous, visceral, retroperitoneal, uterine, gastrointestinal, and vascular LMS, whereas Gladdy et al. expressed results of LMS involving the abdomen, retroperitoneum, trunk wall, and extremities in general. It is worth mentioning here that amongst these three studies, the exclusion criteria of Farshid et al. most closely resemble our exclusion criteria. They found that a positive margin (intralesional or marginal) was the only significant factor associated with metastases, and the margin status strongly correlated with larger size and deeper location. Lung metastases were commonest followed by the liver and skin. Furthermore, the results of Svavar et al. suggested that higher grade, large tumor size, and deeper location were independent predictors of significantly decreased metastasis-free survival. These findings agree with the overall results.

The study by Gladdy et al. found that grade and size were independent predictors of both metastasis and survival. Location seems to be the factor which most authors identify as an independent predictor of metastasis [6]. In contrast to Farshid et al., our analysis did not show any correlation between the type of excision and metastasis development in the follow-up period [4].

Few authors have explored the impact of neoadjuvant therapy in treating LMS. Radiotherapy has shown an effect on disease progression [7, 13] and survival [13]. There is limited evidence on the use of chemotherapy in LMS and its influence on the outcome. With limited data we had on adjuvant therapy, our analysis showed that adjuvant chemotherapy or adjuvant radiotherapy had no influence on survival. However, adjuvant radiotherapy was an independent factor in reducing the risk of metastasis.

Finally, even though many studies have consistently reported LMS to be an aggressive soft tissue tumor with increased risk of local recurrence and metastasis, there have been few studies which have explored the impact of various clinical and pathological factors affecting local recurrence. Despite there being two studies [6, 7] which did explore the development of local recurrence as the outcome, there was insufficient site-specific data or data exclusive to local recurrence, to enable collation and analysis.

The strength of our study lies in the fact that the outcome variables have been characterized in a manner that allows for pooling of the data and computation of the results. An additional strength of this study lies in the fact that studies included in the systematic review have had multivariate analyses performed on similar prognostic factors, making the synchronization and pooling methodology used in this study reliable. However, given the relatively small sample sizes included in the study, it is possible that some prognostic factors may not have become significant and may have been undetected. To overcome this, we have used the nonsignificant results to make our estimates more precise. In summary, we have tried to collect all the data that was available to us irrespective of statistical significance.

We have also calculated the OR statistically using the data from the study to avoid looking into only those prognostic factors which appear to be significant. Hence, we believe that our methods have reduced the risk of calculating an overestimated risk from these publications. In doing this, we have tried to overcome the “outcome bias.”

One might argue that the existing risk prediction models like “PERSARC”^20^ or “Sarculator” have studied the risk factors for soft tissue sarcomas in general and provided prediction tools. Findings from these articles like radiotherapy leading to better outcome, age having an adverse outcome on overall survival, and increasing tumor size having a worsening prognosis for local recurrence and overall survival are similar to some of our findings. We believe each pathological entity is distinct in their behavior, and our study will enable us to comprehensively understand the risk factors associated with LMS’s poor outcomes. Furthermore, this will allow future investigators to investigate the role of adjuvant treatment or enhanced follow-up to improve the outcome of high-risk group patients with LMS of trunk wall and extremities.

A significant concern and the major limitation of our study are the heterogeneity of the studies and that of the reported data. In particular, the differences in cutoff values for the various factors by the authors make the pooled results less reliable. Specifically, the tumor grading, to some extent to age, size, location of LMS, usage of chemotherapy, and data on recurrence, was heterogeneously reported. We have attempted to harmonize the data to make them more acceptable using random-effect analysis. Despite this, the general methodical heterogeneity might have hampered the pooling of study results [18].

## Conclusions

Our study shows that size larger than 5 cm and positive excision margin may be independent prognostic factors associated with the risk of death. From the available information in the literature, one may assume that margins of excision, size, and deep location could be independent prognostic factors increasing the risk of local recurrence. On the other hand, limited evidence on the influence of adjuvant treatment suggested that radiotherapy may be an independent factor in reducing the risk of metastasis. There is a need for methodically high-quality studies with more uniform study design and reporting to evaluate the impact of various risk factors on local recurrence and metastases. Furthermore, collaborative work involving specialized centers and multicenter cooperation will improve the situation and eventually enable more accurate individual prognostication.

## Supplementary Information

**Additional file 1.** PRISMA Checklist.

**Additional file 2.** Full Search Strategy.

**Additional file 3.** Study quality assessment according to Newcastle-Ottawa Scale.

**Additional file 4.** Assessment of quality of studies based on Hayden at al 2006.

## Data Availability

All data generated or analyzed during this study are included in this published article.

## Abbreviations

CI: Confidence interval
FNCLCC: French Federation of Cancer Centers Sarcoma Group
HR: Hazard ratio
LMS: Leiomyosarcoma
OR: Odds ratio
PRISMA: Preferred Reporting Items for Systematic Reviews and Meta-Analyses
RT: Radiotherapy
SMA: Smooth muscle actin
STS: Soft tissue sarcomas
