# Supplementary material for: Leiomyosarcoma in the extremities and trunk wall: systematic review and meta-analysis of the oncological outcomes
Source: World J Surg Oncol. 2022 Apr 18;20:124. doi: 10.1186/s12957-022-02584-4 (PMC9014567; doi:10.1186/s12957-022-02584-4)
Supplement: Supplementary file 2 — Additional file 2. Full Search Strategy. [file 12957_2022_2584_MOESM2_ESM.docx]

**Literature Search – Strategy 1**

1. Medline; leiomyosarcoma.ti,ab;

2. Medline; LEIOMYOSARCOMA/;

3. Medline; 1 OR 2;

4. Medline; recur*.ti,ab;

5. Medline; RECURRENCE/;

6. Medline; NEOPLASM RECURRENCE, LOCAL/;

7. Medline; 4 OR 5 OR 6;

8. Medline; 3 AND 7;

16. Medline; (upper adj2 (limb OR extremit*)).ti,ab;

17. Medline; (lower adj2 (limb OR extremit*)).ti,ab;

18. Medline; UPPER EXTREMITY/;

19. Medline; LOWER EXTREMITY/;

20. Medline; (arm OR leg).ti,ab;

21. Medline; exp ARM/;

22. Medline; exp LEG/;

23. Medline; 16 OR 17 OR 18 OR 19 OR 20 OR 21 OR 22;

25. Medline; metasta*.ti,ab;

26. Medline; NEOPLASM METASTASIS/;

27. Medline; 25 OR 26;

28. Medline; (trunk AND wall).ti,ab;

29. Medline; TORSO/;

30. Medline; 28 OR 29;

31. Medline; 5 OR 6 OR 27;

32. Medline; 23 OR 30;

34. EMBASE; LEIOMYOSARCOMA/;

35. EMBASE; leiomyosarcoma.ti,ab;

36. EMBASE; 34 OR 35;

37. EMBASE; RECURRENT DISEASE/;

38. EMBASE; CANCER RECURRENCE/ OR TUMOR RECURRENCE/ OR METASTASIS/;

39. EMBASE; metasta*.ti,ab;

40. EMBASE; recur*.ti,ab;

41. EMBASE; 37 OR 38 OR 39 OR 40;

42. EMBASE; (upper adj2 (limb OR extemit*)).ti,ab;

43. EMBASE; (lower adj2 (limb OR extemit*)).ti,ab;

44. EMBASE; exp LEG/;

45. EMBASE; exp ARM/;

46. EMBASE; (arm OR leg).ti,ab;

47. EMBASE; (trunk AND wall).ti,ab;

48. EMBASE; TRUNK/;

49. EMBASE; 42 OR 43 OR 44 OR 45 OR 46 OR 47 OR 48;

51. EMBASE; 36 AND 41 AND 49;

52. Medline; 3 and 31 and 32;

53. EMBASE,Medline; Duplicate filtered: [36 AND 41 AND 49], [3 and 31 and 32];

**Literature Search – Strategy 2**

1. Medline; leiomyosarcoma.ti,ab;

2. Medline; LEIOMYOSARCOMA/;

3. Medline; 1 OR 2;

4. Medline; recur*.ti,ab;

5. Medline; RECURRENCE/;

6. Medline; NEOPLASM RECURRENCE, LOCAL/;

7. Medline; 4 OR 5 OR 6;

8. Medline; 3 AND 7;

17. Medline; metasta*.ti,ab;

18. Medline; NEOPLASM METASTASIS/;

19. Medline; 17 OR 18;

25. EMBASE; LEIOMYOSARCOMA/;

26. EMBASE; leiomyosarcoma.ti,ab;

27. EMBASE; 25 OR 26;

28. EMBASE; RECURRENT DISEASE/;

29. EMBASE; CANCER RECURRENCE/ OR TUMOR RECURRENCE/ OR METASTASIS/;

30. EMBASE; metasta*.ti,ab;

31. EMBASE; recur*.ti,ab;

32. EMBASE; 28 OR 29 OR 30 OR 31;

33. Medline; (predict* adj3 outcome*).ti,ab;

34. Medline; "OUTCOME ASSESSMENT (HEALTH CARE)"/;

35. Medline; 33 OR 34;

36. Medline; 7 OR 19;

37. Medline; 3 AND 35 AND 36;

38. EMBASE; (predict* adj3 outcome*).ti,ab;

39. EMBASE; OUTCOME ASSESSMENT/;

40. EMBASE; 38 OR 39;

41. EMBASE; 27 AND 32 AND 40;

42. Medline,EMBASE; Duplicate filtered: [3 AND 35 AND 36], [27 AND 32 AND 40];

**Literature Search – Strategy 3**

1. Medline; leiomyosarcoma.ti,ab;

2. Medline; LEIOMYOSARCOMA/;

3. Medline; 1 OR 2;

4. Medline; recur*.ti,ab;

5. Medline; RECURRENCE/;

6. Medline; NEOPLASM RECURRENCE, LOCAL/;

7. Medline; 4 OR 5 OR 6;

8. Medline; 3 AND 7;

17. Medline; metasta*.ti,ab;

18. Medline; NEOPLASM METASTASIS/;

19. Medline; 17 OR 18;

25. EMBASE; LEIOMYOSARCOMA/;

26. EMBASE; leiomyosarcoma.ti,ab;

27. EMBASE; 25 OR 26;

28. EMBASE; RECURRENT DISEASE/;

29. EMBASE; CANCER RECURRENCE/ OR TUMOR RECURRENCE/ OR METASTASIS/;

30. EMBASE; metasta*.ti,ab;

31. EMBASE; recur*.ti,ab;

32. EMBASE; 28 OR 29 OR 30 OR 31;

33. Medline; (predict* adj3 outcome*).ti,ab;

34. Medline; "OUTCOME ASSESSMENT (HEALTH CARE)"/;

35. Medline; 33 OR 34;

36. Medline; 7 OR 19;

37. Medline; 3 AND 35 AND 36;

38. EMBASE; (predict* adj3 outcome*).ti,ab;

39. EMBASE; OUTCOME ASSESSMENT/;

40. EMBASE; 38 OR 39;

41. EMBASE; 27 AND 32 AND 40;

42. Medline,EMBASE; Duplicate filtered: [3 AND 35 AND 36], [27 AND 32 AND 40];

43. EMBASE; BIOLOGICAL MARKER/ OR DISEASE MARKER/;

44. EMBASE; ((clinical OR molecular OR immunological) adj2 marker).ti,ab;

45. EMBASE; MOLECULAR MARKER/;

46. EMBASE; TUMOR MARKER/;

47. EMBASE; 43 OR 44 OR 45 OR 46;

48. EMBASE; 27 AND 32 AND 47;

49. Medline; BIOMARKERS/;

50. Medline; (clinical AND marker*).ti,ab;

51. Medline; ((molecular OR immunological) adj2 marker).ti,ab;

52. Medline; 49 OR 50 OR 51;

53. Medline; 3 AND 36 AND 52;

54. EMBASE,Medline; Duplicate filtered: [27 AND 32 AND 47], [3 AND 36 AND 52];

55. EMBASE; ((Univariate OR multivariate) AND analysis).ti,ab;

56. EMBASE; 27 AND 32 AND 55;

57. Medline; ((Univariate OR multivariate) AND analysis).ti,ab;

58. Medline; 3 AND 36 AND 57;

59. EMBASE,Medline; Duplicate filtered: [27 AND 32 AND 55], [3 AND 36 AND 57];

**Literature Search – Updated Search from 2020**

1. EMBASE; trunk*.ti,ab;

2. EMBASE; extremi*.ti,ab;

3. EMBASE; soft tissue*.ti,ab;

4. EMBASE; leiomyosarc*.ti,ab;

5. EMBASE; sarcoma*.ti,ab;

6. EMBASE; 1 OR 2 OR 3;

7. EMBASE; 4 OR 5;

8. EMBASE; 6 AND 7;

9. MEDLINE; trunk*.ti,ab;

10. MEDLINE; ; extremi*.ti,ab;

11. MEDLINE ; soft tissue*.ti,ab;

12. MEDLINE; leiomyosarc*.ti,ab;

13. MEDLINE; sarcoma*.ti,ab;

14. MEDLINE; 9 OR 10 OR 11;

15. MEDLINE; 12 OR 13;

16. MEDLINE; 14 AND 15;
