# Supplementary material for: Leiomyosarcoma in the extremities and trunk wall: systematic review and meta-analysis of the oncological outcomes
Source: World J Surg Oncol. 2022 Apr 18;20:124. doi: 10.1186/s12957-022-02584-4 (PMC9014567; doi:10.1186/s12957-022-02584-4)
Supplement: Supplementary file 3 — Additional file 3. Study quality assessment according to Newcastle-Ottawa Scale. [file 12957_2022_2584_MOESM3_ESM.docx]

**Supplementary 2:** Study quality assessment according to Newcastle-Ottawa Scale.

| **Author** | **Represen-tativeness** | **Sample size** | **Non-responders** | **Exposure** | **Compara-bility** | **Assessm-ent** | **Statistics** | **Selection score out of 5** | **Compara-bility score out of 2** | **Outcome score out of 3** | **Total out of 10** |
| --- | --- | --- | --- | --- | --- | --- | --- | --- | --- | --- | --- |
| Hashimoto et al 1995  [17] | 1 | 0 | 1 | 2 | 2 | 1 | 0 | 4 | 2 | 2 | 8 |
| Gustafson et al 1992  [2] | 1 | 0 | 1 | 2 | 2 | 1 | 0 | 4 | 2 | 2 | 8 |
| Miyajima et al 2002  [11] | 1 | 0 | 1 | 2 | 1 | 1 | 0 | 4 | 1 | 2 | 7 |
| Farshid  et al 2002  [4] | 1 | 0 | 1 | 2 | 1 | 1 | 0 | 4 | 1 | 2 | 7 |
| Massi  et al 2004  [10] | 1 | 0 | 1 | 2 | 2 | 1 | 0 | 4 | 2 | 2 | 8 |
| Svarvar  et al 2006  [14] | 1 | 0 | 1 | 2 | 2 | 1 | 0 | 4 | 2 | 2 | 8 |
| Tsiatis  et al 2009  [19] | 1 | 0 | 1 | 2 | 1 | 1 | 0 | 4 | 1 | 2 | 7 |
| Abraham  et al 2012  [15] | 1 | 0 | 1 | 2 | 1 | 1 | 0 | 4 | 1 | 2 | 7 |
| Gladdy  et al 2013  [7] | 1 | 0 | 1 | 2 | 2 | 1 | 0 | 4 | 1 | 2 | 8 |
| Farid  et al 2013  [1] | 1 | 0 | 1 | 2 | 2 | 1 | 0 | 4 | 1 | 2 | 8 |
| Gordon  et al 2014  [18] | 1 | 0 | 1 | 2 | 2 | 1 | 0 | 4 | 1 | 2 | 8 |
| Worhunsky  et al 2015  [5] | 1 | 0 | 1 | 2 | 1 | 1 | 0 | 4 | 1 | 2 | 7 |
| Shoushtari  et al 2016  [16] | 1 | 0 | 1 | 2 | 1 | 1 | 0 | 4 | 1 | 2 | 7 |
