# Supplementary material for: Leiomyosarcoma in the extremities and trunk wall: systematic review and meta-analysis of the oncological outcomes
Source: World J Surg Oncol. 2022 Apr 18;20:124. doi: 10.1186/s12957-022-02584-4 (PMC9014567; doi:10.1186/s12957-022-02584-4)
Supplement: Supplementary file 4 — Additional file 4. Assessment of quality of studies based on Hayden at al 2006. [file 12957_2022_2584_MOESM4_ESM.docx]

**Supplementary 3:** Assessment of quality of studies based on Hayden at al 2006.

| **Author** | **Study participation** | **Study attrition** | **Prognostic measurement** | **Outcome measurement** | **Confounding measurement** | **Analysis** | **Score out of 7 (Yes= 1, No= 0, Partly= 0.5)** |
| --- | --- | --- | --- | --- | --- | --- | --- |
| Hashimoto et al 1995  [17] | Yes | Yes | Yes | Yes | Yes | No | 6 |
| Gustafson et al 1992  [2] | Yes | Yes | Yes | Yes | Yes | Yes | 7 |
| Miyajima et al 2002  [11] | Yes | Yes | Yes | Partly | Partly | Yes | 6 |
| Farshid  et al 2002  [4] | Yes | Yes | Yes | Yes | Yes | Yes | 7 |
| Massi  et al 2004  [10] | Yes | Yes | Yes | Yes | Yes | Yes | 7 |
| Svarvar  et al 2006  [14] | Yes | Yes | Yes | Yes | Yes | Yes | 7 |
| Tsiatis  et al 2009  [19] | Yes | Yes | Partly | Partly | Partly | Yes | 5.5 |
| Abraham  et al 2012  [15] | Yes | Yes | Yes | Yes | Yes | Partly | 6.5 |
| Gladdy  et al 2013  [7] | Yes | Yes | Yes | Yes | Yes | Partly | 6.5 |
| Farid  et al 2013  [1] | Yes | Yes | Yes | Yes | Yes | Yes | 7 |
| Gordon  et al 2014  [18] | Yes | Yes | Yes | Yes | Yes | Yes | 7 |
| Worhunsky  et al 2015  [5] | Yes | Yes | Yes | Partly | Partly | Yes | 6 |
| Shoushtari  et al 2016  [16] | Yes | Yes | Yes | Partly | Partly | Yes | 6 |
